# Association between outdoor artificial light at night and metabolic diseases in middle-aged to older adults—the CHARLS survey

**DOI:** 10.3389/fpubh.2025.1515597

**Published:** 2025-03-06

**Authors:** Mingyuan Fan, Jiushu Yuan, Sai Zhang, Qingqing Fu, Dingyi Lu, Qiangyan Wang, Hongyan Xie, Hong Gao

**Affiliations:** ^1^Hospital of Chengdu University of Traditional Chinese Medicine, Chengdu, Sichuan, China; ^2^Department of Endocrinology, Hospital of Chengdu University of Traditional Chinese Medicine, Chengdu, China; ^3^TCM Regulating Metabolic Diseases Key Laboratory of Sichuan Province, Hospital of Chengdu University of Traditional Chinese Medicine, Chengdu, China

**Keywords:** China Health and Retirement Longitudinal Study, metabolic diseases, artificial light at night, environmental factors, circadian rhythms

## Abstract

**Introduction:**

Artificial light at night (LAN) is associated with metabolic diseases, but its precise relationship is still not fully understood. This study explores the association between LAN and metabolic diseases.

**Methods:**

A cross-sectional study involving 11,729 participants conducted in 2015 was selected from the China Health and Retirement Longitudinal Study. Diabetes, metabolic syndrome (MetS), overweight, obesity, dyslipidemia, and hyperuricemia (HUA) were defined according to established guidelines. Using satellite data, we estimated LAN exposure for 2015 and matched each participant’s address with the corresponding annual mean LAN value. Multivariate logistic regression models were used to assess the relationship between LAN and metabolic diseases. To investigate possible non-linear associations and visualize the dose–response relationship between LAN and metabolic diseases, we used the restricted cubic splines (RCS) regression model.

**Results:**

We found that higher levels of LAN significantly correlate with metabolic diseases. In the final adjusted model, participants in the highest LAN quartile group (Q4) showed the highest risk for metabolic diseases: diabetes [odds ratio (OR): 1.03, 95% confidence interval (CI): 1.01, 1.05], MetS (OR: 1.04, 95% CI: 1.02, 1.06), overweight (OR: 1.08, 95% CI: 1.06, 1.11), obesity (OR: 1.03, 95% CI: 1.01, 1.05), and dyslipidemia (OR: 1.03, 95% CI: 1.01, 1.05). In the RCS regression model, there was a non-linear association between LAN and risk of MetS, overweight, obesity, dyslipidemia, and HUA (for non-linear: *p* < 0.05).

**Conclusion:**

LAN is associated with an increased risk of metabolic diseases. This highlights the urgent need to address LAN pollution in public health strategies; reducing LAN exposure may help mitigate the risk of metabolic diseases.

## Highlights

- Outdoor artificial LAN is positively associated with metabolic diseases.
- Outdoor artificial LAN could be a potential risk factor for metabolic diseases.
- Highlighting the need for further research to investigate the long-term effects of outdoor artificial LAN on metabolic health.

## 1 Introduction

The prevalence of metabolic diseases, including diabetes, obesity, and hyperlipidemia, is surging globally, posing a major challenge to global public health (1, 2). Identifying risk factors for preventive interventions is essential to effectively address this issue. While genetic and lifestyle factors are well documented, emerging evidence suggests that environmental factors, particularly outdoor artificial light at night (LAN), may play a key role.

In recent decades, rapid urbanization has significantly illuminated the human nighttime environment, with over 80% of the global population exposed to LAN (3). Night work and the extensive use of electronic devices have further increased human exposure to LAN, disrupting circadian rhythms (4). This disruption impacts the endogenous biological clock, altering sleep–wake patterns, hormone secretion, and numerous physiological processes within the endocrine system. Circadian rhythms are crucial for maintaining metabolic homeostasis, and their disruption may elevate the risk of several metabolic diseases, including diabetes and obesity (5). Despite the increasing attention to LAN pollution, its potential impact on metabolic diseases is still largely underexplored.

This study aimed to investigate the correlation between LAN and the risk of developing diabetes, being overweight, obesity, dyslipidemia, hyperuricemia (HUA), and metabolic syndrome (MetS) using the China Health and Retirement Longitudinal Study (CHARLS) data. By enhancing our understanding of the relationship between environmental light exposure and metabolic health, this research emphasizes the importance of incorporating LAN into metabolic disease prevention strategies. Ultimately, these findings may encourage the public to take action to reduce light pollution and mitigate the health effects of LAN.

## 2 Methods

### 2.1 Data source and analytical sample

This study utilized data from CHARLS 2015—a prospective study involving adults aged ≥45 years and older across 28 provinces in China. Ethical approval for the CHARLS was reviewed by the Peking University Institutional Review Board (IRB), and all participants provided informed consent before enrollment (6). A total of 11,719 participants provided baseline information, underwent physical examination, submitted fasting blood samples, and participated in relevant assessments. The inclusion process is shown in Supplementary Figure S1.

### 2.2 Assessment of outdoor artificial LAN

The data for the Chinese administrative area were obtained from the National Platform for Common GeoSpatial Information Services.^1^ It provides the most recent vertical data for 2024 pertaining to China’s prefectural and municipal administrative regions in the GCS_WGWS_1984 coordinate system. LAN values were obtained from the nighttime light remote sensing dataset similar to the Defense Meteorological Satellite Program Operational Linescan System (DMSP-OLS) (7). This dataset employs the “pseudo-invariant pixel” method to calibrate the DMSP-OLS data and correct the missing data in the original monthly Suomi National Polar-Orbiting Partnership Visible Infrared Imaging Radiometer Suite (SNPP-VIIRS) data before synthesizing the annual data. We calculated mean annual LAN values for each study location (prefecture-level city) using calibrated DMSP-OLS-like data. Using the city associated with each participant’s identity (ID), we employed ArcGIS 10.8.1 to produce mean annual LAN exposure values for each participant. The original dataset can be accessed through the Harvard Dataverse platform.^2^

### 2.3 Definition of metabolic diseases

#### 2.3.1 Diabetes

Diabetes was diagnosed based on fasting blood glucose levels ≥126 mg/dL, glycosylated hemoglobin ≥6.5% (8), use of glucose-lowering medication, or a previous diagnosis by a physician.

#### 2.3.2 Overweight and obesity

Body mass index (BMI) was calculated by dividing weight (in kg) by height (in m^2^). According to Chinese standards, a BMI of 24.0–27.9 kg/m^2^ is classified as overweight, and a BMI of ≥28.0 kg/m^2^ is classified as obese, respectively (9).

#### 2.3.3 Dyslipidemia

Dyslipidemia was defined as triglycerides (TG) ≥ 150 mg/dL, total cholesterol (TC) ≥ 200 mg/dL, low-density lipoprotein cholesterol (LDL-C) ≥ 130 mg/dL, or high-density lipoprotein cholesterol (HDL-C) < 40 mg/dL in men and < 50 mg/dL in women (10). A physician’s notification regarding a medical history or the use of lipid-lowering medications also indicated dyslipidemia.

#### 2.3.4 Hyperuricemia

HUA was defined as a serum uric acid level of ≥7.0 mg/dL in men and ≥ 6.0 mg/dL in women (11).

#### 2.3.5 Metabolic syndrome

MetS was defined as meeting at least three of the following five criteria: Central obesity (waist circumference: men ≥85 cm, women ≥80 cm), elevated TG (≥150 mg/dL, or treatment), reduced HDL-C (<40 mg/dL in men, <50 mg/dL in women, or treatment), elevated blood pressure (systolic ≥140 mm Hg and/or diastolic ≥90 mm Hg, or treatment), and elevated fasting glucose (≥100 mg/dL, or treatment) (12).

### 2.4 Covariates

Age (<65 years or ≥ 65 years), gender (female or male), education (primary school or lower, secondary school, or university degree or higher), marital status (married or single), residence (urban or rural), smoking status (non-smoker or smoker), and alcohol consumption status (non-consumer of alcohol, consumes alcohol but less than once a month, consumes alcohol more than once a month), and daily living ability [activities of daily living (ADL) and instrumental activities of daily living (IADL)] were considered in the analysis. The ADL assessment included dressing, bathing, bedtime routines, eating, urinating, and defecating. Any task that requires assistance is considered to have an ADL disability. The IADL assessment included housework, cooking, shopping, managing finances and medications, and making phone calls. Any task that requires assistance is considered as having an IADL disability.

### 2.5 Statistical analysis

Continuous variables that follow a normal distribution are represented as means and standard deviations (SDs). Continuous variables with anomalous distribution are expressed as the median [interquartile range (IQR)]. Categorical variables are expressed as percentages. Since the LAN was left-skewed, we categorized it into quartile groups (Q1, Q2, Q3, and Q4), using the lowest quartile group (Q1) as the reference group. To assess the relationship between LAN and metabolic diseases, multivariate logistic regression models were developed for analysis using LAN as either a continuous (per IQR increment) or categorical (quartiles) variable. When model 1 was adjusted for age, model 2 was further adjusted for gender, education, marital status, smoking status, consuming alcohol status, residence, ADL, and IADL. The results are presented as odds ratios (ORs) with 95% confidence intervals (CIs). We also examined the possible non-linear relationship between LAN and metabolic diseases using restricted cubic spline (RCS) regression. All statistical analyses were performed using R 4.3.3. A two-sided *p*-value of less than 0.05 indicates statistical significance.

## 3 Results

### 3.1 Outdoor LAN distribution in 2015

**Figure 1:**
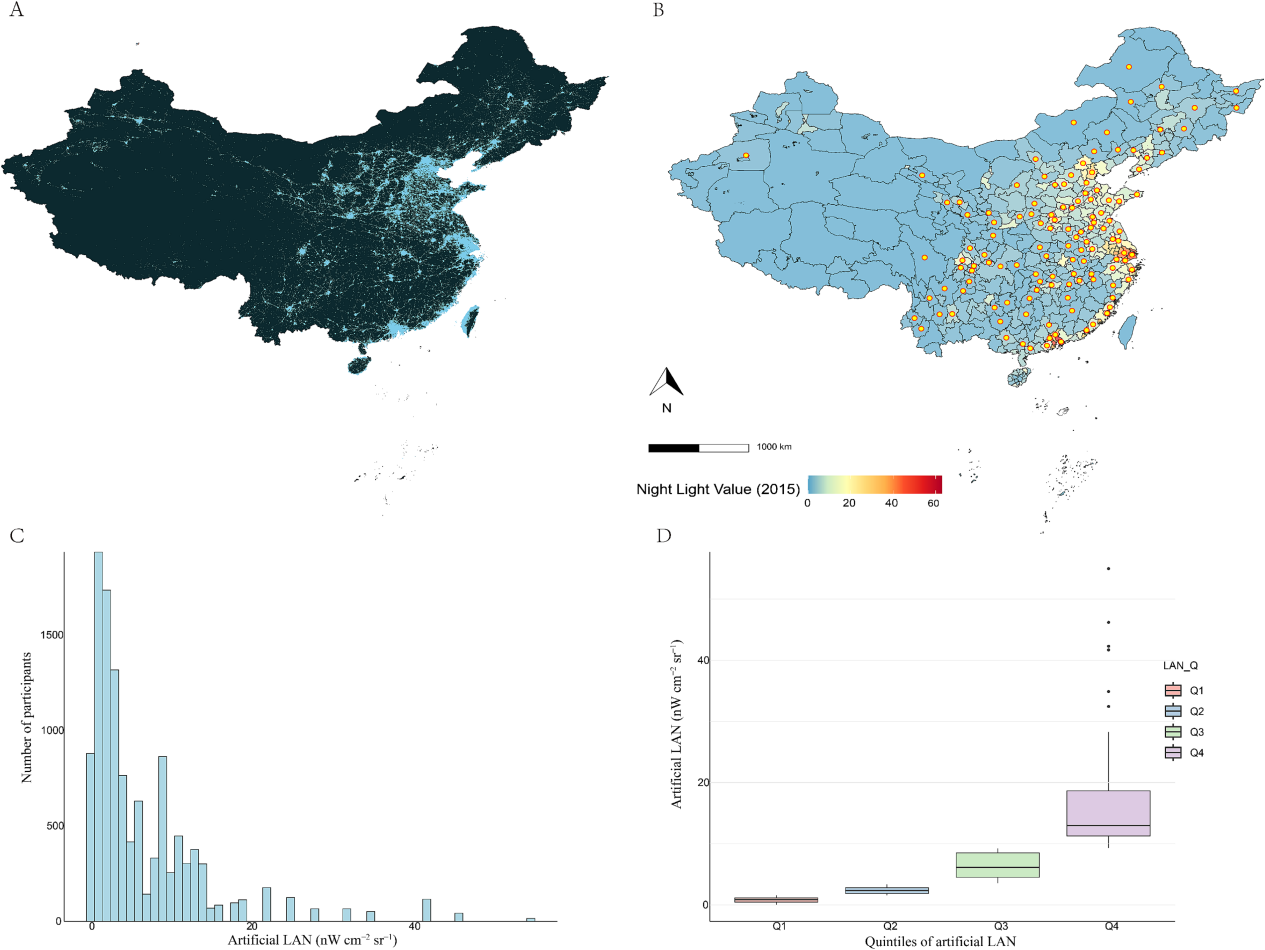
Distribution of outdoor artificial LAN exposure. **(A,B)** Map of outdoor artificial LAN in China in 2015, with study sites indicated by circles. **(C)** Number of participants exposed to each LAN value. **(D)** Radiation distribution by outdoor artificial LAN quartiles. The median (25% quartile, 75% quartile) of LAN in quintile 1 to quintile 4, respectively, was 0.89 (0.48, 1.17), 2.37 (1.89, 2.83), 6.15 (4.53, 8.52), and 13.0 (11.3, 18.7) nWcm^−2^ sr^−1^.

Outdoor artificial LAN exposure in 2015 varied extensively across China (Figures 1A–C). The majority of the areas were exposed to low-intensity outdoor artificial LAN, while higher-intensity exposure was concentrated in eastern coastal cities. The distribution of LAN across the 125 study sites was left-skewed, with a median (IQR) LAN exposure of 3.37 (1.57, 9.25) nWcm^−2^ sr^−1^.

### 3.2 Study population characteristics

**Figure 2:**
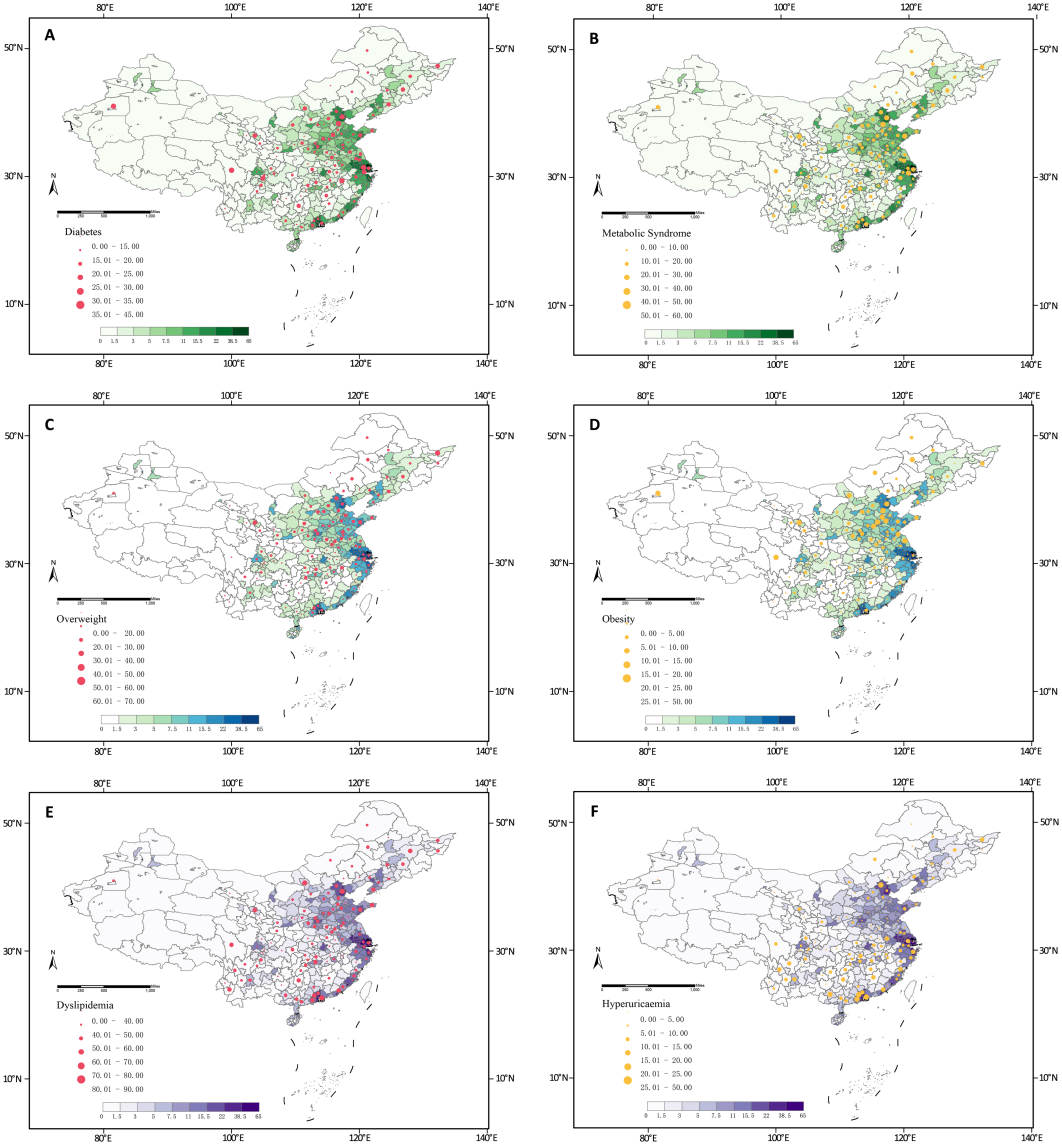
Spatial distribution of LAN exposure and incidence of metabolic diseases. **(A)** Spatial distribution of LAN exposure and incidence of diabetes. **(B)** Spatial distribution of LAN exposure and incidence of metabolic syndrome. **(C)** Spatial distribution of LAN exposure and incidence of overweight.**(D)** Spatial distribution of LAN exposure and incidence of obesity. **(E)** Spatial distribution of LAN exposure and incidence of dyslipidemia. **(F)** Spatial distribution of LAN exposure and incidence of hyperuricaemia.

This study included 11,729 participants, with a mean age of 60.3 ± 9.5 years. In subsequent analyses, we categorized participants into quartiles of outdoor artificial LAN. The median (IQR) for quartiles 1 through 4 was 0.89 (0.48,1.17), 2.37 (1.89, 2.83), 6.15 (4.53, 8.52), and 13.0 (11.3, 18.7) nWcm^−2^ sr^−1^, respectively (Figure 1D). Table 1 presents the baseline characteristics of participants exposed to LAN. Participants with higher LAN exposure were older, had a higher BMI, and were more likely to live in urban areas. Specifically, the Q4 group had a mean age of 60.3 years and a BMI of 24.5, in contrast to the Q1 group’s mean age of 59.5 years and a BMI of 23.4. With regard to educational attainment, 79.5% of the Q4 group possessed a college degree or higher, while this figure was 76.0% in the Q1 group. Furthermore, 19.7% of the Q4 group lived in urban areas compared to 11.9% of the Q1 group. In contrast, participants in the Q1 group reported higher levels of singleness (12.6% in Q1 vs. 11.0% in Q4), smoking prevalence (33.4% vs. 29.3%), and frequency of alcohol consumption (more than once a month: 27.4% vs. 26.6%). Among the 125 study sites, cities with higher metabolic disease rates were concentrated in areas with higher LAN exposure. Figure 2 presents the spatial distribution of LAN exposure and metabolic disease rates across all study sites. Specifically, the prevalence of metabolic diseases tended to be higher in participants with larger LAN exposure: diabetes (23.0% in Q4 vs. 17.9% in Q1), MetS (32.5% vs. 24.0%), overweight (37.6% vs. 28.4%), obesity (15.2% vs. 11.6%), dyslipidemia (59.3% vs. 53.3%), and HUA (13.1% vs.10.7%).

**Table 1:** Participants’ characteristics by quintiles of outdoor artificial LAN exposure.

	Overall (*N* = 11,729)	Q1 (*N* = 2,977)	Q2 (*N* = 2,892)	Q3 (*N* = 3,077)	Q4 (*N* = 2,783)	*P*
BMI (kg/m^2^)	24.0 (4.0)	23.4 (3.9)	23.7 (3.9)	24.5 (4.1)	24.5 (3.8)	<0.001
Age (years)	60.3 (9.5)	59.5 (9.2)	61.1 (9.7)	60.5 (9.4)	60.3 (9.5)	<0.001
LAN [mean (SD)]	6.5 (7.8)	0.8 (0.4)	2.4 (0.5)	6.3 (1.9)	17.2 (9.2)	<0.001
Age group						
<65 years	7,904 (67.4)	2059 (69.2)	1871 (64.7)	2044 (66.4)	1930 (69.3)	<0.001
≥65 years	3,825 (32.6)	918 (30.8)	1,021 (35.3)	1,033 (33.6)	853 (30.7)	
Gender						
Female	6,162 (52.5)	1,554 (52.2)	1,494 (51.7)	1,611 (52.4)	1,503 (54.0)	0.324
Male	5,567 (47.5)	1,423 (47.8)	1,398 (48.3)	1,466 (47.6)	1,280 (46.0)	
Educational level						
Primary school or lower	2021 (17.2)	588 (19.8)	457 (15.8)	557 (18.1)	419 (15.1)	<0.001
Secondary school	678 (5.8)	127 (4.3)	162 (5.6)	237 (7.7)	152 (5.5)	
University degree or higher	9,030 (77.0)	2,262 (76.0)	2,273 (78.6)	2,283 (74.2)	2,212 (79.5)	
Marital status						
Married	10,264 (87.5)	2,603 (87.4)	2,497 (86.3)	2,688 (87.4)	2,476 (89.0)	0.028
Single	1,465 (12.5)	374 (12.6)	395 (13.7)	389 (12.6)	307 (11.0)	
Residence						
Rural	9,802 (83.6)	2,622 (88.1)	2,387 (82.5)	2,557 (83.1)	2,236 (80.3)	<0.001
Urban	1927 (16.4)	355 (11.9)	505 (17.5)	520 (16.9)	547 (19.7)	
Smoking status						
Non-smoker	8,078 (68.9)	1983 (66.6)	1985 (68.6)	2,142 (69.6)	1968 (70.7)	0.006
Smoker	3,651 (31.1)	994 (33.4)	907 (31.4)	935 (30.4)	815 (29.3)	
Drinking status						
Non-drinker	7,537 (64.3)	1879 (63.1)	1833 (63.4)	2012 (65.4)	1813 (65.1)	0.346
Drink but less than once a month	1,036 (8.8)	283 (9.5)	258 (8.9)	266 (8.6)	229 (8.2)	
Drink more than once a month	3,156 (26.9)	815 (27.4)	801 (27.7)	799 (26.0)	741 (26.6)	
ADL_disability (%)	565 (4.8)	181 (6.1)	152 (5.3)	141 (4.6)	91 (3.3)	<0.001
IADL_disability (%)	1936 (16.5)	554 (18.6)	500 (17.3)	528 (17.2)	354 (12.7)	<0.001
Central obesity	7,503 (64.0)	1,669 (56.1)	1722 (59.5)	2,139 (69.5)	1973 (70.9)	<0.001
Elevated serum triglycerides	3,847 (32.8)	934 (31.4)	946 (32.7)	981 (31.9)	986 (35.4)	0.005
Reduced serum HDL-C	112 (1.0)	28 (0.9)	20 (0.7)	35 (1.1)	29 (1.0)	0.329
Elevated blood pressure	4,677 (39.9)	1,131 (38.0)	1,067 (36.9)	1,334 (43.4)	1,145 (41.1)	<0.001
Elevated plasma glucose	4,390 (37.4)	958 (32.2)	1,107 (38.3)	1,145 (37.2)	1,180 (42.4)	<0.001
Diabetes	2,451(20.9)	534 (17.9)	608 (21.0)	669 (21.7)	640 (23.0)	<0.001
Metabolic syndrome	3,279 (28.0)	714 (24.0)	742 (25.7)	919 (29.9)	904 (32.5)	<0.001
Overweight	3,937 (33.6)	845 (28.4)	930 (32.2)	1,116 (36.3)	1,046 (37.6)	<0.001
Obesity	1,582 (13.5)	344 (11.6)	313 (10.8)	503 (16.3)	422 (15.2)	<0.001
Dyslipidemia	6,496 (55.4)	1,587 (53.3)	1,538 (53.2)	1722 (56.0)	1,649 (59.3)	<0.001
Hyperuricaemia	1,339 (11.4)	320 (10.7)	344 (11.9)	310 (10.1)	365 (13.1)	0.002

In summary, higher exposure to LAN was associated with distinct demographic, educational, and health characteristics. Participants in the highest quartile of LAN exposure were older, had a higher BMI, more frequently lived in urban areas, achieved higher educational levels, and demonstrated different health behaviors and disease prevalence compared to those in the lowest quartile.

### 3.3 LAN exposure and metabolic diseases association

**Figure 3:**
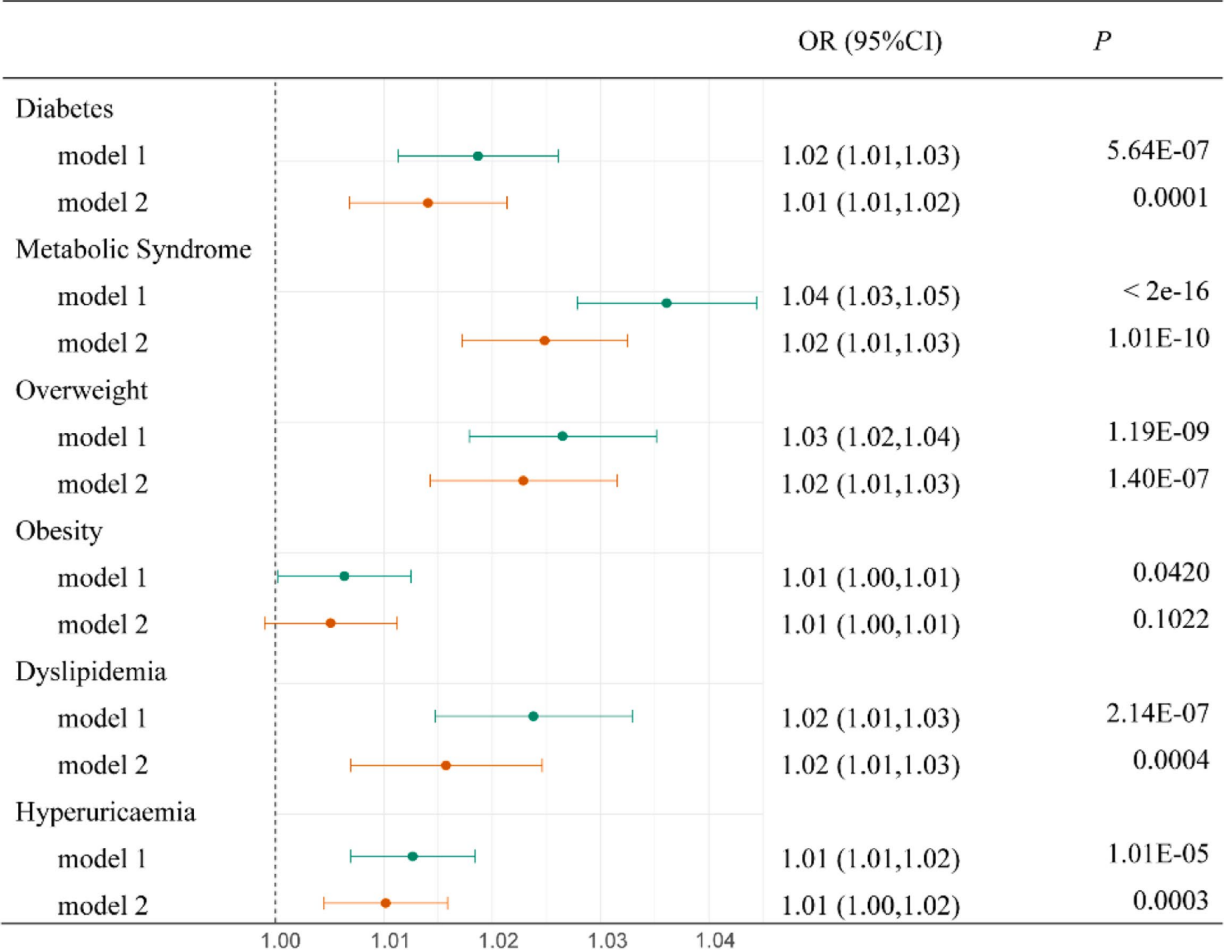
Association of LAN IQR with metabolic diseases. Model 1: Adjusted for age. Model 2: Based on Model 1, further adjustments for sex, education, marital status, smoking status, alcohol consumption, residence, ADL, and IADL.

**Figure 4:**
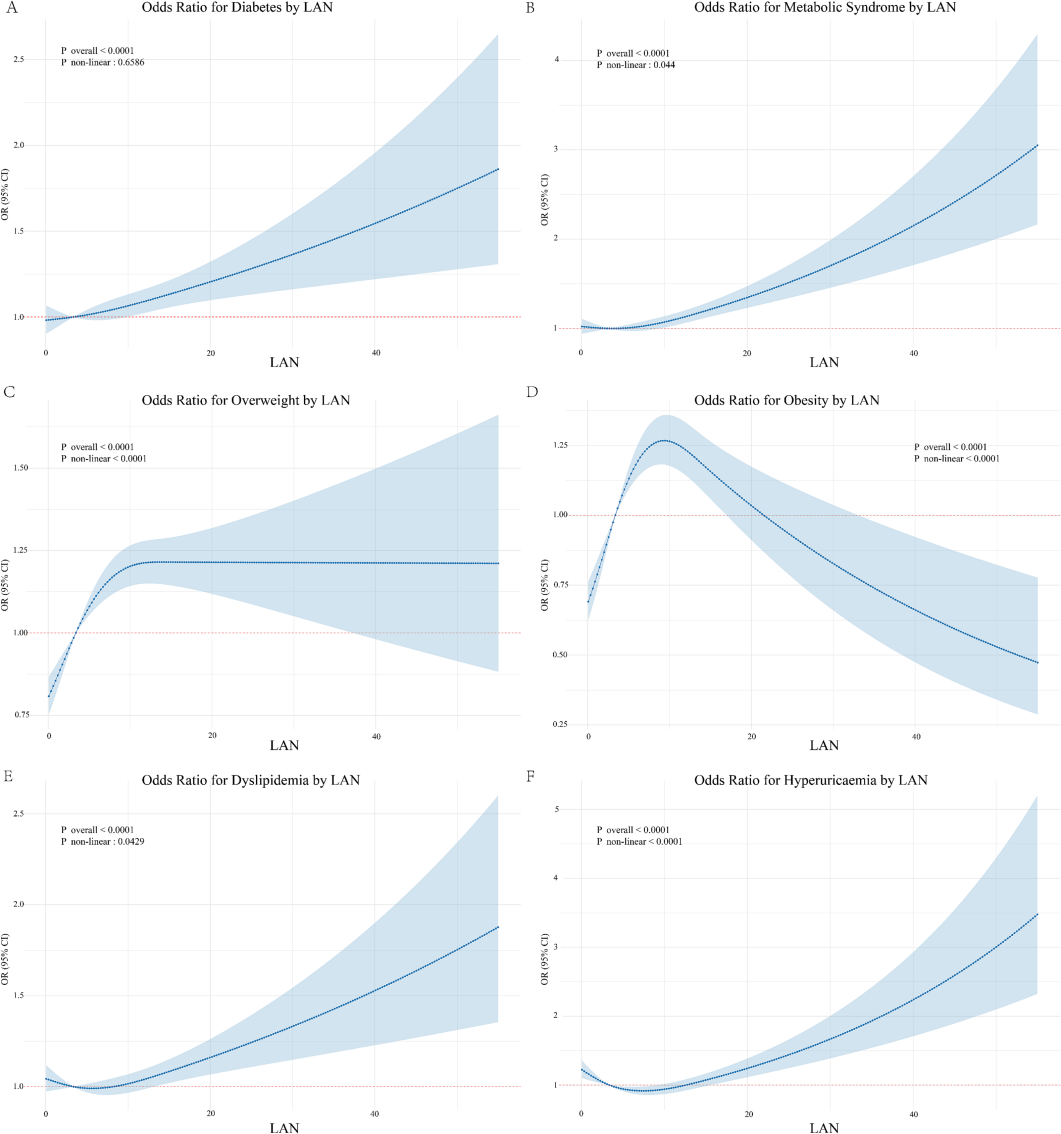
Spline curves for LAN and metabolic diseases **(A)** Spline curves for LAN and diabetes. **(B)** Spline curves for LAN and metabolic syndrome. **(C)** Spline curves for LAN and overweight.**(D)** Spline curves for LAN and obesity. **(E)** Spline curves for LAN and dyslipidemia. **(F)** Spline curves for LAN and hyperuricaemia.

In the final adjusted model (Figure 3), LAN was significantly associated with an elevated risk of diabetes (OR: 1.01, 95% CI: 1.01, 1.02), MetS (OR: 1.02, 95% CI: 1.01, 1.03), overweight (OR: 1.02, 95% CI: 1.01, 1.03), dyslipidemia (OR: 1.02, 95% CI: 1.01, 1.03), and HUA (OR: 1.01, 95% CI: 1.00, 1.02). Table 2 shows the association between LAN quartiles and the risk of metabolic diseases. In the final adjusted model, the risk of diabetes, MetS, overweight, obesity, and dyslipidemia increased progressively with increasing LAN quartiles (for trend, *p* < 0.05). Compared to the Q1 group, the Q4 group developed diabetes (OR: 1.03, 95% CI: 1.01, 1.05), MetS (OR: 1.04, 95% CI: 1.02, 1.06), overweight (OR: 1.08, 95% CI: 1.06, 1.11), obesity (OR: 1.03, 95% CI: 1.01, 1.11), and dyslipidemia (OR: 1.03, 95% CI: 1.01, 1.05) had the highest risk. In addition, we examined the shape of the exposure–response curve for the relationship between LAN and metabolic diseases (Figure 4). The RCS regression showed a non-linear (U-shaped) relationship between LAN and risk of MetS, overweight, obesity, dyslipidemia, and HUA (for non-linear, *p* < 0.05).

**Table 2:** Association between LAN quartiles and risk of metabolic diseases.

Quartiles of light	Q1	Q2	Q3	Q4	*P* for trend
Diabetes					
Model 1	Ref	1.03(1.01, 1.05)**	1.04 (1.02, 1.06)***	1.05 (1.03, 1.07)***	1.51e-05 ***
Model 2		1.02 (1.00, 1.04)	1.01 (0.99, 1.04)	1.03(1.01, 1.05)**	0.014*
Metabolic syndrome					
Model 1	Ref	1.02 (0.99, 1.04)	1.06 (1.04, 1.08)***	1.09 (1.06, 1.11)***	5.04e-15 ***
Model 2		1.00 (0.98, 1.02)	1.01 (0.99, 1.03)	1.04 (1.02, 1.06) ***	8.33e-05 ***
Overweight					
Model 1	Ref	1.04 (1.02, 1.07)***	1.08 (1.06, 1.11)***	1.10 (1.07, 1.12)***	3.79e-13 ***
Model 2		1.03(1.01, 1.06)**	1.08 (1.05, 1.10)***	1.08 (1.06, 1.11)***	8.99e-11 ***
Obesity					
Model 1	Ref	1.00 (0.98, 1.01)	1.05 (1.03, 1.07) ***	1.04 (1.02, 1.06) ***	1.57e-07 ***
Model 2		0.99 (0.98, 1.01)	1.05 (1.03, 1.07) ***	1.03(1.01, 1.05) ***	1.22e-06 ***
Dyslipidemia					
Model 1	Ref	1.00 (0.97, 1.03)	1.03(1.00, 1.05)	1.06 (1.03, 1.09)**	1.87e-07 ***
Model 2		0.99 (0.96, 1.01)	0.99 (0.97, 1.02)	1.03(1.01, 1.05)*	0.006**
Hyperuricaemia					
Model 1	Ref	1.01 (0.99, 1.03)	0.99 (0.98, 1.01)	1.02 (1.01, 1.04)**	0.014*
Model 2		1.01 (0.99, 1.02)	0.98 (0.96, 0.99)**	1.01 (1.00, 1.03)	0.286

*p < 0.05, **p < 0.01, ***p < 0.001. Model 1: adjusted for age; Model 2: based on Model 1, further adjustments for gender, education, marital status, smoking status, drinking status, residence, ADL and IADL.

## 4 Discussion

In this study, we found that participants living in areas with higher LAN were more likely to report metabolic diseases. This is, to our knowledge, the first study to use satellite data and a large cohort to examine the association between outdoor artificial LAN radiation and metabolic diseases. We found that higher LAN exposure was positively associated with an increased risk of metabolic diseases. Successive years of LAN growth have led to a spike in light pollution, raising concerns about its potential health hazards. Research indicates that LAN may be associated with metabolic diseases, such as diabetes, obesity, and MetS. The initial observation was that night shift workers were more susceptible to dysglycemia, MetS, and obesity, drawing attention to LAN (13). Studies have confirmed that LAN is a modifiable environmental factor for MetS, and exposure to LAN is significantly associated with a 43–52% increased risk of MetS (14). Chronic exposure to high-intensity outdoor LAN is significantly associated with impaired glucose homeostasis and a higher prevalence of diabetes (15). In older adults, sustained exposure to LAN has been associated with comorbid obesity, diabetes, and hypertension (16), as well as elevated plasma triglycerides, LDL, and reduced HDL (17).

In this study, we observed a significant positive association between outdoor artificial LAN and metabolic diseases, consistent with previous findings. Whether LAN functions as a continuous or categorical variable, it showed a correlation with metabolic diseases, with high levels of LAN indicating the greatest risk. Although the observed ORs for these associations ranged from 1.01 to 1.09, they showed statistically significant correlation consistency. This suggests that even a slight increase in nighttime light exposure can measurably impact metabolic health. The small effect size of the study association may be due to the uneven distribution of LAN in the study population. Higher levels of light pollution are likely more prevalent in economically developed areas along the eastern seaboard, while financially disadvantaged areas may have lower levels of light. We observed that participants with high levels of LAN were more often living in cities. The relatively small percentage of participants residing in urban areas in this study may have impacted the strength of the observed association. The results demonstrate significant statistical significance and consistency, reinforcing previous studies on the effects of LAN on metabolic diseases and highlighting its potential impact on public health.

We observed non-linear associations between LAN exposure and the risks of MetS, overweight, obesity, dyslipidemia, and hyperuricemia. Higher LAN exposure was consistently associated with an increased risk of diabetes, MetS, overweight, dyslipidemia, and hyperuricemia. However, the accuracy of these estimates decreased at high LAN exposure levels, as shown by wider confidence intervals. This reduced precision may result from the left-skewed distribution of LAN, which resulted in a smaller sample size in the high-exposure group. Notably, the U-shaped curve observed for obesity differs from the positive correlation trends seen in other diseases. Specifically, the risk of obesity peaks at moderate LAN exposure levels but decreases at both very low and very high LAN exposure levels. While the relatively small sample size in the high LAN exposure group partially explains this finding, other complex mechanisms may also be involved. For example, higher LAN exposure may correspond to more urbanized areas, where residents often experience healthier lifestyles or better access to medical care, reducing obesity risks despite the rise in nighttime light pollution. Conversely, areas with low LAN exposure may indicate rural or remote regions where residents engage in more physical labor, which could also contribute to a reduced risk of obesity. Future studies should utilize more precise LAN data and larger sample sizes to validate these findings and further investigate the mechanisms underlying the U-shaped relationship between LAN and metabolic diseases. The current mechanistic studies provide insights into the association between LAN and increased risk of metabolic diseases. First, LAN may disrupt circadian rhythms to influence metabolic parameters, both in brain regions and peripheral tissues. Various brain regions contain circadian oscillators driven by central clock genes, which control functions such as sleep, wakefulness, food intake, hormone secretion, and mood, all of which exhibit circadian rhythms (18). LAN interferes with the central clock, leading to circadian disruption in brain regions, translating into a delayed circadian phase of metabolic parameters, which adversely affects glucolipid metabolism (19). In pancreatic islet tissues, mutants of the circadian gene CLOCK and the protein BMAL1 show significantly impaired glucose tolerance, decreased insulin secretion, and pancreatic *β*-cell defects, leading to the onset of diabetes (20). BMAL1/CLOCK promotes circadian rhythm in leptin transcription, mediated by C/EBPα in adipose tissue. Chronic circadian disruption affects the body’s endogenous adipose clock and induces leptin resistance. Dysregulation of the BMAL1/CLOCK coupling may be the key to circadian disruption in the triggering of obesity and MetS (21). Altering food intake timing to gain weight is a key effect of LAN leading to subsequent metabolic dysregulation (22). Food intake is the primary external synchronizer of the peripheral clock. Individuals with disrupted circadian rhythms are more likely to eat late at night, consuming most of their daily energy intake, and late-night eating disrupts the circadian system in a vicious cycle (23). Late-night eating is associated with disturbed daily cortisol rhythms, impaired glucose tolerance, and insulin resistance, which can increase fat accumulation (24). Second, LAN disrupts hormone secretion, particularly by suppressing nocturnal melatonin secretion (25). Melatonin, secreted by the pineal gland, serves as a biological nighttime signal crucial for maintaining metabolic homeostasis and regulating sleep and glucolipid metabolism (26). Melatonin controls the production of the glucose transporter protein GLUT4 and the phosphorylation process of insulin receptors to activate the insulin signaling pathway and maintain glucose homeostasis (27), which may enhance glucose metabolic effects (28). Melatonin supplementation reduces insulin resistance (29) and restores the LAN-damaged hepatic GR/REV-ERBs axis, inhibiting hepatic lipid accumulation (30). Additionally, LAN disrupts the adrenal clock, leading to elevated glucocorticoid levels, potentially due to the targeted deletion of the core clock gene Bmal1 (31). Moreover, LAN affects sleep patterns, increasing the risk of metabolic diseases. Adequate sleep is essential for normal metabolic function, and chronic sleep deprivation, poor sleep quality, and disturbed sleep patterns increase the risk of metabolic diseases (32). Residents in high LAN areas may experience increased sleep deprivation because LAN creates an unfavorable environment that makes individuals more susceptible to disturbances in their sleep patterns. Individuals with altered sleep patterns are often exposed to more LAN, which further disrupts their sleep and reduces sleep duration (33).

In summary, LAN is associated with negative outcomes in metabolic disease. Considering outdoor artificial LAN as a modifiable environmental risk factor may improve metabolic health at the population level. Public health interventions may involve promoting the use of blackout curtains, encouraging the reduction of LAN exposure, and implementing initiatives to reduce light pollution throughout the community. Future research should aim to clarify the causal relationship between LAN and metabolic disease. Longitudinal studies and randomized controlled trials could provide more definitive evidence of the effects of LAN on metabolic health. Our findings emphasize the need to focus on the modifiable environmental factor “LAN” in public health strategies for metabolic diseases. However, because this is a cross-sectional study, future longitudinal studies are necessary to gain a better understanding of the potential long-term effects of LAN exposure on metabolic health.

This study is the first to utilize satellite data and a large longitudinal cohort to investigate the association between outdoor artificial LAN and metabolic disease. One of the main strengths of our study is its large population, which enabled us to explore the associations between individual and environmental characteristics. Furthermore, we adjusted for potential confounders to more accurately characterize the effects of environmental factors. However, our study has some limitations. First, CHARLS lacks access to the exact residential addresses of participants, which may lead to discrepancies in specific nighttime light exposure values. We assumed that participants living in the same city experienced similar exposure levels and calculated average annual LAN values based on this assumption, ensuring a degree of accuracy. Nonetheless, temporal and spatial variations can affect an individual’s actual LAN exposure, particularly during indoor and outdoor nighttime activities. Second, as a cross-sectional study, our findings cannot confirm a causal relationship between LAN and metabolic diseases, requiring further investigation.

## 5 Conclusion

Our study demonstrated a significant association between higher-intensity outdoor artificial LAN exposure and metabolic diseases. Future research should investigate the potentially harmful effects of LAN and explore whether a causal relationship exists between LAN and metabolic diseases, as well as with its underlying mechanisms. This investigation could provide critical evidence for developing effective environmental intervention strategies designed to mitigate metabolic diseases.

## Data Availability

The original contributions presented in the study are included in the article/Supplementary material, further inquiries can be directed to the corresponding author.

## Footnotes

^1^ https://www.tianditu.gov.cn/

^2^ https://dataverse.harvard.edu/dataset.xhtml?persistentId=doi:10.7910/DVN/GIYGJU
