# Supplementary figures and images for: Association between outdoor artificial light at night and metabolic diseases in middle-aged to older adults—the CHARLS survey
Source: Front Public Health. 2025 Mar 6;13:1515597. doi: 10.3389/fpubh.2025.1515597 (PMC11922846; doi:10.3389/fpubh.2025.1515597)

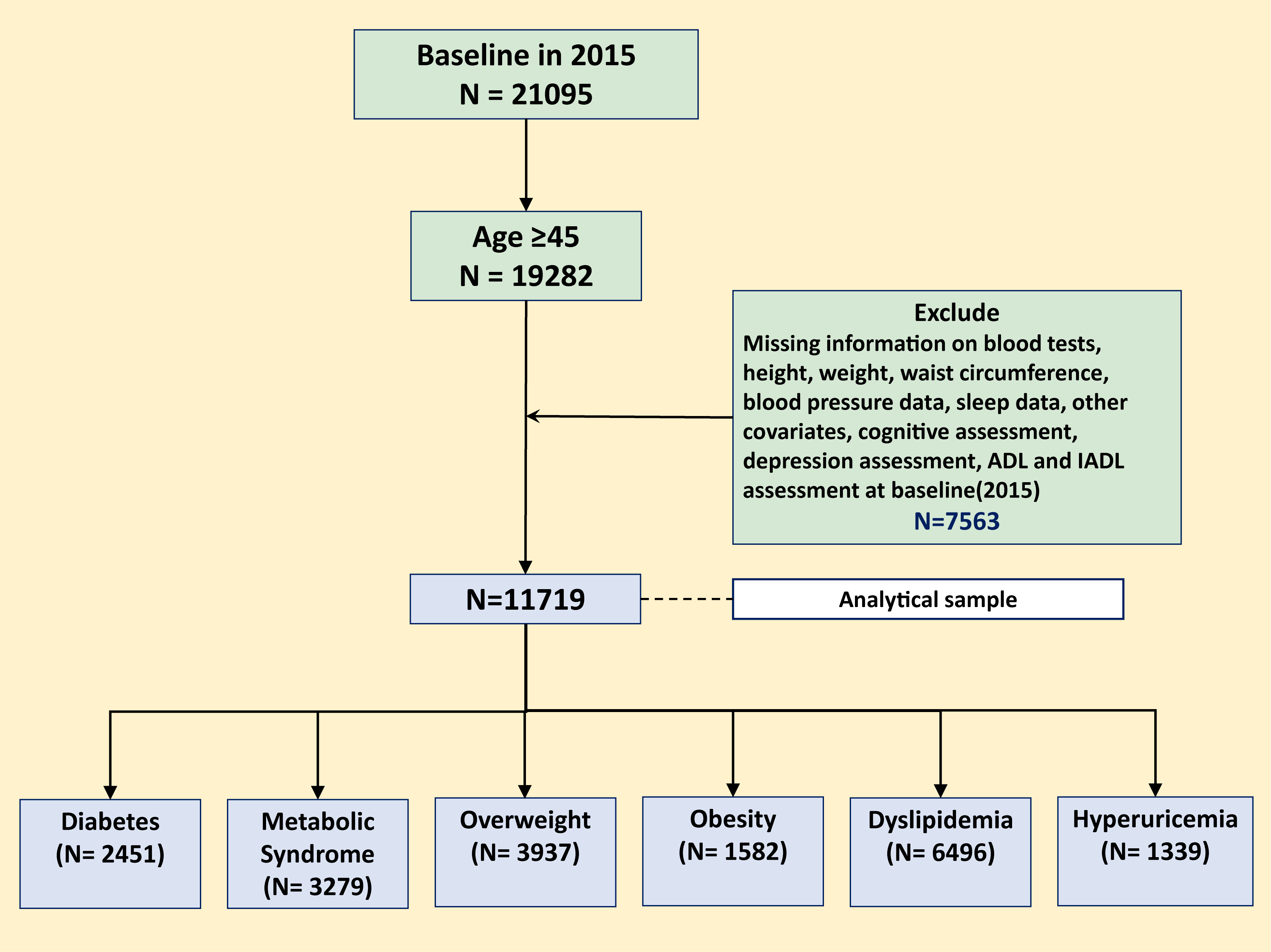

Supplement: Supplementary file 1 [file Image_1.tif]
